# Prior and Posterior Linear Pooling for Combining Expert Opinions: Uses and Impact on Bayesian Networks—The Case of the Wayfinding Model

**DOI:** 10.3390/e20030209

**Published:** 2018-03-20

**Authors:** Charisse Farr, Fabrizio Ruggeri, Kerrie Mengersen

**Affiliations:** 1 School of Mathematical Sciences, Science and Engineering Faculty, Queensland University of Technology, Brisbane, QLD 4000, Australia; 2 Consiglio Nazionale delle Ricerche, Istituto di Matematica Applicata e Tecnologie Informatiche, 20133 Milano, Italy

**Keywords:** bayesian networks, linear pooling, posterior pooling, prior pooling, wayfinding, expert opinions

## Abstract

The use of expert knowledge to quantify a Bayesian Network (BN) is necessary when data is not available. This however raises questions regarding how opinions from multiple experts can be used in a BN. Linear pooling is a popular method for combining probability assessments from multiple experts. In particular, Prior Linear Pooling (PrLP), which pools opinions and then places them into the BN, is a common method. This paper considers this approach and an alternative pooling method, Posterior Linear Pooling (PoLP). The PoLP method constructs a BN for each expert, and then pools the resulting probabilities at the nodes of interest. The advantages and disadvantages of these two methods are identified and compared and the methods are applied to an existing BN, the Wayfinding Bayesian Network Model, to investigate the behavior of different groups of people and how these different methods may be able to capture such differences. The paper focusses on six nodes *Human Factors*, *Environmental Factors*, *Wayfinding*, *Communication*, *Visual Elements of Communication* and *Navigation Pathway*, and three subgroups *Gender* (Female, Male), *Travel Experience* (Experienced, Inexperienced), and *Travel Purpose* (Business, Personal), and finds that different behaviors can indeed be captured by the different methods.

## 1. Introduction

Bayesian networks (BNs) are a popular tool used for describing complicated systems. From their beginnings in computer science, BNs have been increasingly used in a range of fields such as ecology [1], natural resource management [2], computational biology [3], medical diagnosis [4] and forensics [5]. In a large number of these fields, the information required to quantify a BN must be obtained from experts. This in itself has raised methodological questions regarding how the opinions obtained from experts can be introduced into the BN, and how the opinions of multiple experts can be represented.

This paper explores the use of two methods to combine opinions from multiple experts in order to investigate how these methods impact the result of a BN, the different behavior of different groups of people, and how the different methods may be able to capture such differences. This exploration is conducted in the context of a substantive real-world case study of wide interest, notably wayfinding.

A combination of experts’ opinions has received considerable attention in the Bayesian community and many methods have been proposed. The book by [6] is an important reference discussing pros and cons of many approaches, and we refer the interested reader to such book and the references therein. An earlier, thorough review is provided in [7], whereas a more recent contribution is [8]. Pooling methods provide a principled approach to combining expert judgements [6]. In this paper, we concentrate our interest on linear pooling, but other approaches have been presented in literature. Examples include logarithmic pooling, whose properties have been illustrated, e.g., in [9], and a nonlinear geometric combination by [10]. Papers like the latter have the desirable property of external Bayesianity, since the order of posterior updating is irrelevant; that is, combining the experts’ posteriors, after observing data, gives the same results as combining their priors and updating the prior combination with the observed data. Other approaches about combination of experts’ opinions in BNs are possible, like the Measurement Error Approach, proposed in [11], where the opinions at each node are considered as if they were noisy observations of the *true* probability value (i.e., a random effect model). In this paper, we assume that the experts agree on the structure of the BN, i.e., its nodes and their logical relationships, whereas they might have different opinions about probabilities. Pooling of opinions also on the structure of the BN is a different task, beyond the goals of the current paper. The paper is structured as follows. The next section gives an intentionally brief description of Bayesian networks (BNs), with further elaboration throughout the paper. The two pooling methods are then introduced and compared in Section 3. The wayfinding case study, which is based on an existing Wayfinding Bayesian Network Model (WBNM) [12], is introduced in Section 4 and then prior and posterior linear pooling methods are applied in Section 5. Advantages and disadvantages of both approaches are critically discussed in this case study and in the discussion in Section 6.

## 2. Bayesian Networks

**Figure 1:**
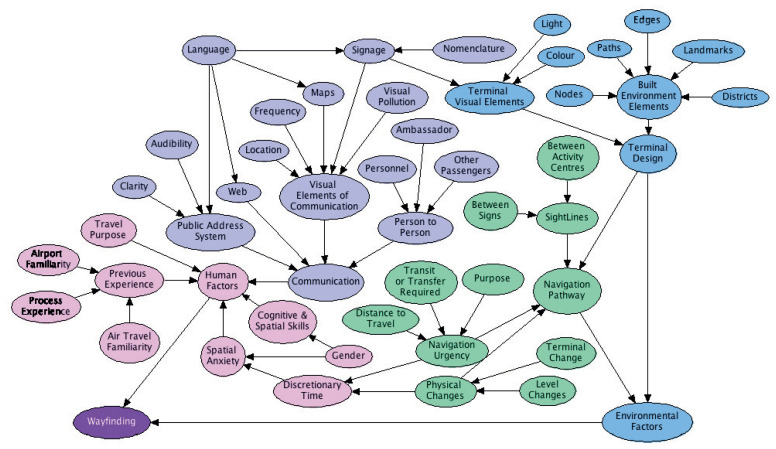
The Wayfinding Bayesian Network [12].

Bayesian networks (BNs) are a graphic modelling method used for reasoning under uncertainty [13]. A BN is constructed as a directed acyclic graph (DAG) that represents variables of interest as nodes and direct dependencies between the variables as directed arrows or arcs [14]. Nodes connected by an arc are commonly called parent or child nodes, depending on the direction of the arrow. An example of this structure is shown later in Figure 1.

In the BNs of interest here, the nodes are discrete in nature, for example Boolean (true or false), ordered values (low, medium, high), and integer ranges (1–49, 50–100), although continuous nodes are also possible [15]. Each node is quantified by a probability table that is either marginal over the states if the node has no parents, or conditional on the states of the parent nodes. These are commonly collectively called conditional probability tables (CPTs) and are populated using data or other information available about the system or problem, including information obtained from experts [15,16]. Each CPT is denoted by P(X${}_{V}|$X${}_{\mathrm{pa}(V)}$), where *V* is a set of one or more nodes in the DAG; P(X${}_{V}$) is the joint probability distributions over the set of variables X${}_{V}$, and X${}_{\mathrm{pa}(V)}$ is the set of parent variables of X${}_{V}$, i.e., those nodes connected directly to *V*. The conditional probabilities take the form $P(X_{V}=x_{V}|X_{\mathrm{pa}(V)}=x_{\mathrm{pa}(V)})=z$. These probabilities thus define a factorisation of a joint probability distribution over the variables represented in the DAG, in that the probability distribution of the BN is the product of the conditional probabilities of each of the variables of a BN conditioned only on its parents [16]. This feature of BNs means that all marginal prior and posterior probabilities can be obtained by marginalizing and conditioning, and that knowledge about one or more variables can be updated as new knowledge or evidence about other variables is acquired [17].

The structure of a BN admits a number of simplifications that can lead to efficiencies in quantification, interpretation and computation. Examples include *d*-separation and the existence of Markov blankets, which are comprised of a node and all the variables that shield it from the rest of the network (i.e., its parents, children and children’s other parents) (see [18,19,20]). Moreover, because they provide a full representation of probability distributions over their variables, they are able to condition upon any subset of their variables and support any direction of reasoning (see [15,16,20]). These include: diagnostic reasoning from symptom to cause, following the opposite direction to network links; predictive reasoning from new information about causes to new beliefs about effects, following the direction of the network links; and intercausal reasoning about the mutual causes of a common effect, following a *v*-structure in the network.

Some of the practical advantages of BNs are that they are suitable for small and incomplete datasets; that they allow for structural learning; that different sources of knowledge and data types can be combined; that they can be solved analytically and hence can provide a fast, real-time response to queries; and that they can be extended to incorporate spatial and temporal dynamic processes [21,22,23].

## 3. Linear Pooling Methods for Combining Opinions

A common way in which to combine the probabilities $P_{i}\left(X\right)$ obtained from experts is linear pooling [24]. In its most general form, the probabilities required are calculated by $P\left(X\right)=\sum_{i=1}^{n}w_{i}P_{i}\left(X\right)$, where $w_{i}$ are positive weights given to each of the *n* experts and $\sum_{i=1}^{n}w_{i}=1$. In this paper, each of the *n* experts is given equal weighing since the wayfinding process is a person-specific experience. Since the purpose of the WBNM was to investigate the factors that influence or impact wayfinding in airports for all users, each respondent’s experience was considered to be equal in value. Hence, $w_{i}=1/n$ and so $P\left(X\right)=\sum_{i=1}^{n}P_{i}\left(X\right)/n$.

*Prior Linear Pooling* (PrLP), the most common pooling method, pools the elicited probabilities within each node. These probabilities are then propagated through the single network to find the marginal probabilities for the nodes of interest. *Posterior Linear Pooling* (PoLP) uses the elicited probabilities to form a BN for each of the *n* experts. Pooling is then undertaken at the final node, or at particular nodes of interest, to obtain the relevant marginal probability distribution. The idea of PoLP has been around for a while in the BN community, but the authors are unaware of papers that formalized such approach. In both approaches, pooling is performed just at individual nodes, not jointly in more nodes: in this way, we are avoiding the general problem that linear pools in multivariate settings do not preserve, in general, independence.

### Advantages and Disadvantages of Linear Pooling Methods for Combining Opinions

**Figure 2:**
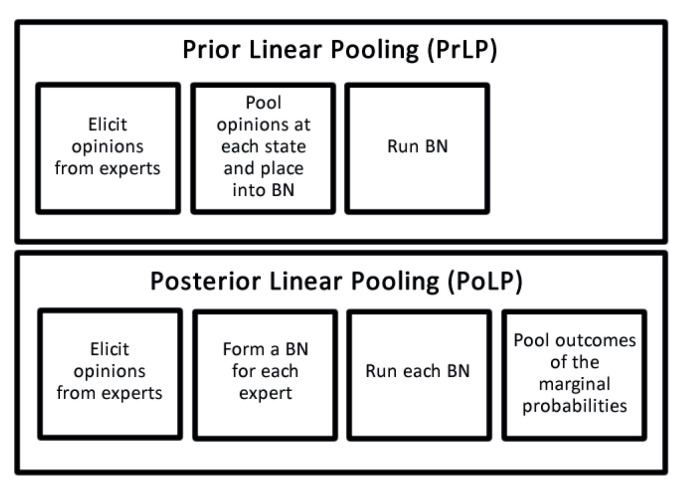
The steps involved in the expert opinion combination methods used.

Each method, of course, has its advantages and disadvantages. The first difference is the number of steps required to get to the stage where the final marginal probabilities of the nodes in the BN can be found. As shown in Figure 2, PrLP only requires three steps before information about the marginal probabilities can be obtained. This differs from PoLP, which requires an extra step to obtain marginal probability information about the nodes of interest. This is because the latter method requires *n* BNs (where *n* is the number of experts) to be formed before it can be used.

PrLP allows the single BN, which contains the pooled opinions at each state, to be used straight away for diagnostic, predictive, and intercausal [15] reasoning. It also allows the updating of information in the BN to be done quickly, which is one of the advantages of using BNs. The number of BNs required for each method makes the model reasoning a more cumbersome process for PoLP. Reasoning and information updating with this method needs to be done for each BN, and then the pooling method is applied. Depending on the number of updates, reasoning types, and BNs, this could become a time-consuming task. A list of advantages and disadvantages for each method is shown in Table 1. Further discussion of the issues raised in the table is deferred to Section 6.

To investigate the impact of the different methods on the outcome of a BN, and to see if behaviors of subgroups of different groups of respondents can be found as a result of using the different methods, they are applied here to an existing BN, the Wayfinding Bayesian Network Model [12].

## 4. The Wayfinding Bayesian Network Model

The Wayfinding Bayesian Network Model (WBNM) [12] was developed to investigate the factors that influence effective wayfinding in airports. The model brings together two factors required for wayfinding: human and environmental. Previous research had been split into these two district streams: human factors such as cognition, memory and spatial recognition were investigated by cognitive scientists [26,27,28,29,30,31]; environmental factors such as the number of sight lines and visual connectivity between activity centres were modelled using mathematical measures such as the Visibility Index (VI) [32,33,34] and inter-connection density (ICD) [35]. The WBNM was applied to the airport setting to investigate the factors that contributed to effective wayfinding in airports. Of particular interest to this paper is that the WBNM was quantified using expert opinion, obtained through a series of surveys. This makes it an ideal BN to investigate the impact of different methods of combining opinions from multiple experts, the behaviors of groups of people, and how the PrLP and PoLP may be able to capture these differences.

The WBNM [12] shown in Figure 1 contains 49 nodes and 58 connections. This BN was used to investigate the factors that influence effective wayfinding in an airport. This model brought together the human and environmental aspects of wayfinding into one model. Previous wayfinding research was split into investigating the human factors involved in wayfinding such as memory, cognitive mapping, spatial recognition, and information processing [26,27,28] or by using index measures that were heavily reliant on the environmental factors associated with the process. These measures included the Inter-Connection Density, which measures the complexity of a floor plan [35], and the Visibility Index, which gives a measure of the ease of wayfinding to the value of available sight lines in an environment [32,33,34].

The development of the model was undertaken with the feedback from focus groups comprised of a multi-disciplinary team with varying levels of air travel and airport experience, a thorough review of the current wayfinding research [36], and feedback from airport operators and BN modelers [12]. The model was quantified using a combination of data obtained from focus groups, wayfinding literature, and an online survey. The online survey was redeployed and the 99 responses are what will be used in this case study.

## 5. Case Study: Linear Pooling Methods and the Wayfinding Bayesian Network Model

To make the evaluation of the Linear Pooling methods easier, a subset of the nodes from the WBNM were chosen. The nodes chosen were *Communication*, *Environmental Factors*, *Human Factors*, *Navigation Pathway*, *Visual Elements of Communication*, and *Wayfinding*. The definition and the states for these nodes are shown in Table 2 [36]. *Communication*, *Navigation Pathway*, and *Visual Elements of Communication* were chosen for the number of parents that they have, as well as their interest for airport operators. The other nodes were chosen as they are nodes of interest for the wayfinding problem. The subgroups of interest chosen are *Gender* (Female, Male), *Travel Experience* (Experienced, Inexperienced), and *Travel Purpose* (Business, Personal).

### 5.1. Prior Linear Pooling (PrLP)

Using Prior Linear Pooling, the elicited probabilities from the 99 experts were pooled and entered into the CPTs for each node of the WBNM. The resulting marginal probabilities for the six nodes of interest showed that there is no change in the marginal probabilities for *Communication*, *Environmental Factors*, *Navigation Pathway*, and *Visual Elements of Communication* across the three subgroups of interest when compared with the results from the full model. This is due to the structure of the network as these nodes are not immediate parents or children of any of the nodes associated with the subgroups of interest. The remaining nodes, *Human Factors* and *Wayfinding*, were found to be heavily effected by *Gender* and *Travel Experience*. The impact of *Gender* (particularly *Females*) is higher on *Human Factors*, whereas *Travel Experience* (particularly *Inexperienced*) has a higher impact on *Wayfinding* effectiveness. Table 3 shows the marginal probabilities for *Human Factors* and *Wayfinding*, with the first row, ‘All’, showing the results from compiling the BN. The probabilities in the remaining rows are found by changing the states of the subgroups of interest. This kind of model interrogation is a strength of BNs and is easy to do. Using this strength, the four possible combinations of *Gender* and *Experience* were used and propagated through the network. It was found that *Inexperienced Female* travelers caused the greatest change in the marginal probabilities for *Human Factors* and *Wayfinding* than any of the other combinations (Table 4).

Another strength of BNs is their ability to provide full representations of probability distributions over the nodes in the network meaning that they can be conditioned upon any subset of the nodes in the network. This allows any direction of reasoning to occur [15]. Since a single BN is used in Prior Linear Pooling, different kinds of reasoning are able to be completed quickly and easily. For example, *diagnostic reasoning*, that is, reasoning from symptom to cause can be performed if, for example, *Wayfinding* observed to be 100% effective. In this situation, the *Effective* state on the *Wayfinding* node would be set to 100%, the other nodes of interest would update, and the updated marginal probabilities can be found. Investigating this diagnostic reasoning on the three subgroups of interest shows that, in order to obtain this situation, Good *Human Factors* need to increase by 0.1337 for *Females*, 0.1554 for *Males*, 0.1487 for *Experienced* travelers, 0.1267 for *Inexperienced* travelers, and 0.1454 for *Business* and *Personal* travel. The change required in *Human Factors* is an order of magnitude larger than the change required for any of the other nodes (Table 5). It can be argued that this may be the case since *Human Factors* is a parent node to *Wayfinding*; however, this argument does not explain why *Environmental Factors*, which is also a parent node, does not result in the same increase. This result indicates that, in order for *Wayfinding* to be 100% effective, a large increase in good *Human Factors* is required.

Using PrLP for combining expert opinions for use in BNs is convenient, as the single BN used allows for model interrogation, scenario testing, reasoning, and what-if analysis to be undertaken quickly. One disadvantage of this method that has not previously been mentioned is that information regarding the basic descriptive statistics of the responses for each probability is ignored. This type of information may provide useful information for inference, and may provide additional information on subgroups of interest. Posterior Linear Pooling is a method that may be able to capture this information.

### 5.2. Posterior Linear Pooling (PoLP)

PoLP develops a BN for each expert and then pools the resulting marginal probabilities at particular nodes of interest. In order to investigate the impact of this pooling method on the WBNM and in particular the nodes of interest, posterior pooling was first run on the full network. That is, the 99 BNs obtained from the survey responses were propagated individually and the results pooled at the six nodes of interest, with the resulting marginal probabilities noted for each of these nodes. The process was then repeated for each subgroup. The number for each subgroup were 46 Females, 53 Males, 32 travelers whose travel purpose was Business, 67 travelers whose travel purpose was Personal, 85 Experienced travelers, and 14 Inexperienced travelers.

The marginal probabilities for the full network for the nodes of interest and the corresponding marginal probabilities for each of the subgroups are shown in Table 6. It can be seen that there are differences between and within each subgroup when compared with the results from the full model for each of the nodes of interest. The absolute difference between the results of the full model and each subgroup for each node of interest can be seen in the square brackets of the same table. The nodes of interest whose marginal probabilities had the largest difference were *Human Factors* and *Wayfinding*. This supports the earlier results from the PrLP analysis of the same BN. Interestingly, PrLP shows that the subgroup *Inexperienced* had the largest difference in marginal probabilities across the six nodes of interest.

**Figure 3:**
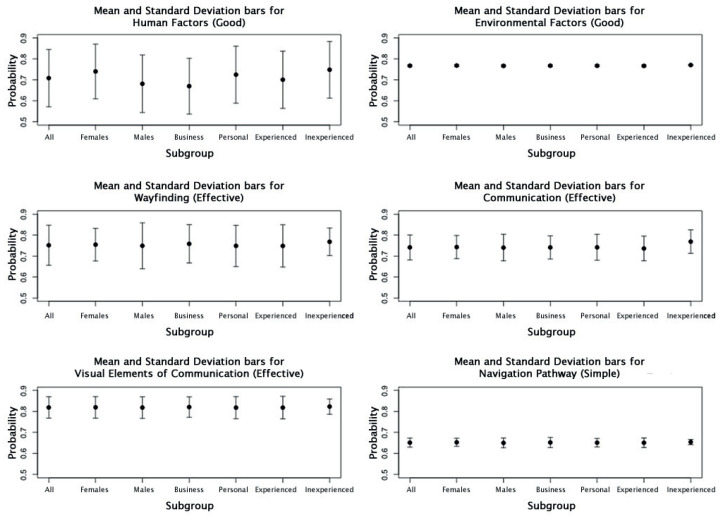
Mean and Standard Deviation bar plots for the subgroups *Gender*, *Travel Purpose*, and *Travel Experience*, grouped for each node of interest.

Since PoLP provides *n* BNs (where *n* is the number of respondents) before pooling occurs, it is possible to investigate the spread of the responses for each subgroup. Figure 3 shows the mean and standard deviation bars for the entire response group and for each subgroup of interest, for the six nodes of interest. The node with the largest standard deviation across all subgroups was *Human Factors*. Interestingly, the spread in this node is consistent across all subgroups for all the respondents. The node with the smallest standard deviation was *Environmental Factors*, followed by *Navigation Pathway*. This may be due to the fact that, while the factors associated with the environment of an airport are important, a greater importance is placed on *Human Factors*. In addition, perhaps most travelers have the same level of expectation of what the environmental factors in an airport should be. The mean for *Human Factors* is visibly different across all subgroups and to the mean for all responses for the node. This is different from the mean responses for each subgroup across the other nodes, which look to be consistent with the mean of all the responses for each respective node. From Figure 3, the *Wayfinding* node, which looks to have a consistent mean across all subgroups, is the only node where the standard deviation bars do not have the same length.

**Figure 4:**
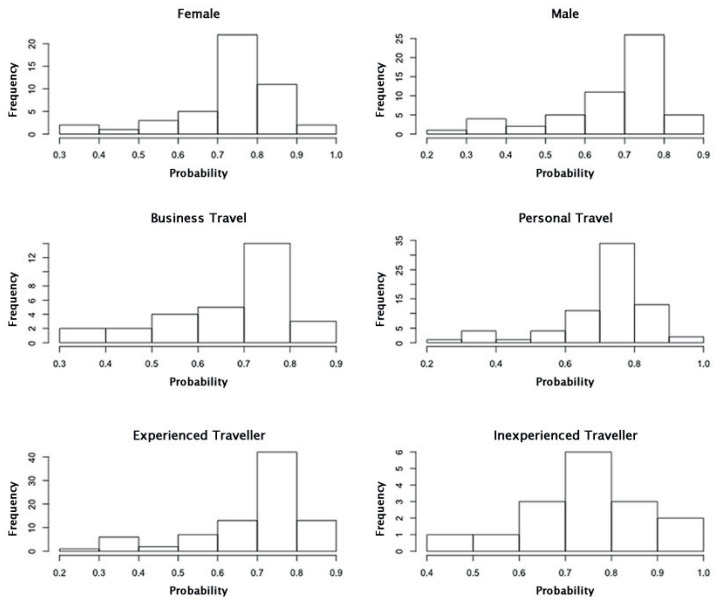
Histograms of the responses from the subgroups of interest for the node *Human Factors*, in the state *Good*.

**Figure 5:**
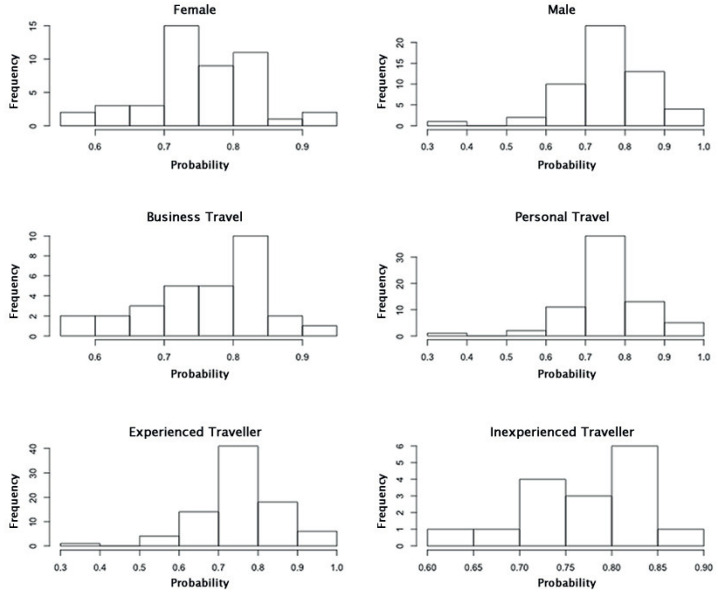
Histograms of the responses from the subgroups of interest for the node *Wayfinding*, in the state *Effective*.

Since *Human Factors* had the most spread and varying mean, a histogram of the responses from each subgroup for this node was plotted. Figure 4 shows that the distribution of responses are approximately normal with most of the responses centred around 0.7–0.8, across all subgroups, and with a spread from 0.2 to 1.0. A similar set of plots (Figure 5) for *Wayfinding* shows a similar distribution behavior across all subgroups, with the exception of *Inexperienced Traveler*. The range of responses is less consistent, ranging from 0.6 to 1.0 for *Female*, *Business Travel*, and *Inexperienced Traveler* and 0.3–1.0 for *Male*, *Personal Travel*, and *Experienced Traveler*. This may explain the varying standard deviation bars for this node, as seen in Figure 3.

**Figure 6:**
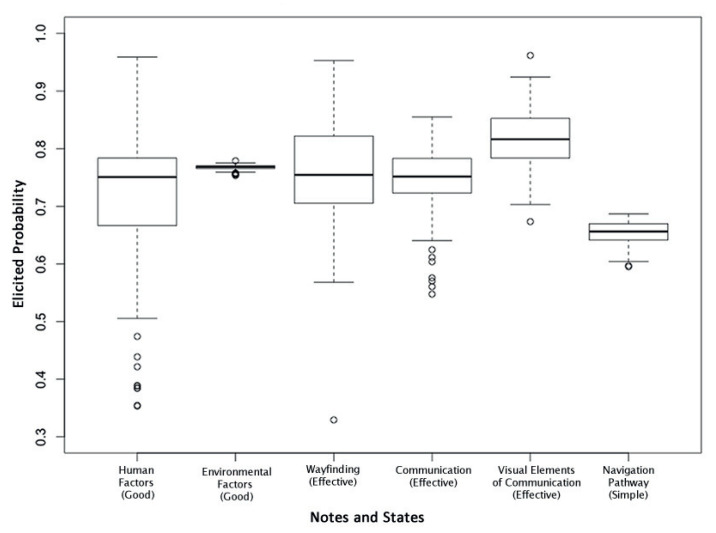
Boxplot showing the spread of responses for the six nodes of interest. For simplicity, only one state per node is shown since the states are binary.

**Figure 7:**
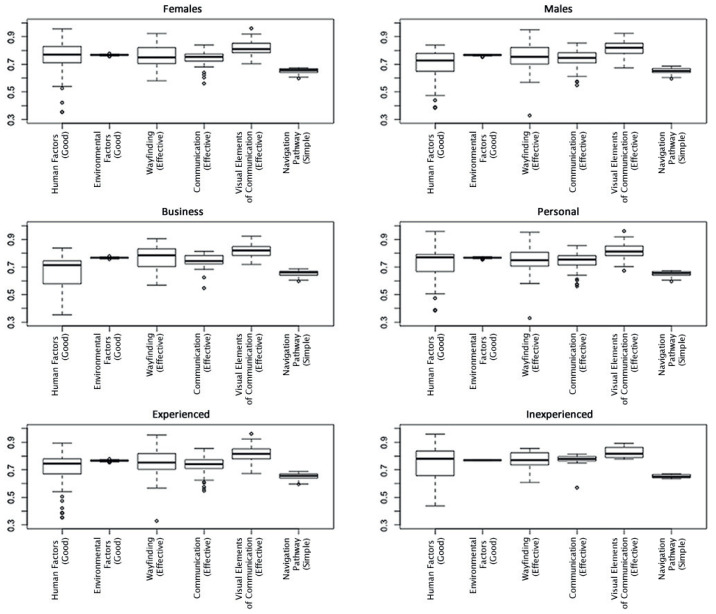
Boxplots of the probabilities for each node of interest, grouped per subgroup.

To further investigate the responses given by the subgroups for each node of interest, boxplots were constructed. Figure 6 shows the spread of responses for the six nodes of interest, with the widest spread associated with *Human Factors* followed by *Wayfinding*. *Environmental Factors* and *Navigation Pathway* has the narrowest spread of responses. By deconstructing these results and grouping the results for each subgroup, as shown in Figure 7, it can be seen that *Inexperienced* travelers reported higher probabilities of *Good Environmental Factors* and *Effective Communication* in order to effectively find their way around an airport. It is also worth noting that the spread of responses by these travelers was much narrower for the other nodes when compared to the other subgroups. The spread of responses for *Effective Wayfinding*, *Good Visual Elements of Communication* and *Simple Navigation Pathway* was generally similar across all subgroups; however, it was much narrower for the *Inexperienced* subgroup. Interestingly, the *Females*, *Personal*, and *Inexperienced* groups required *Human Factors* to be higher for the same level of *Wayfinding*. Conversely, it can be seen that those in the *Males*, *Business* and *Experienced* groups have a narrower range for *Human Factors* for the same level of *Wayfinding* effectiveness to occur.

**Figure 8:**
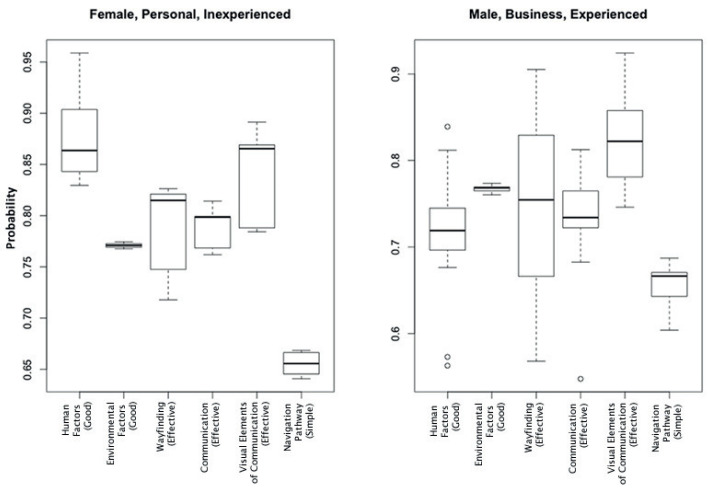
Boxplots showing the spread of responses for each node of interest for *Female, Inexperienced Travelers* on *Personal Travel*, and for *Experienced Male Travelers* traveling for *Business*. For simplicity, only one state per node is shown since the states are binary.

The data from the BNs were organized into the eight possible combinations for the three subgroups to see what impact these combinations had on the nodes of interest. Based on the observations from Figure 7, plots for *Female, Personal, Inexperienced* and *Male, Business, Experienced* were generated (Figure 8). Both subgroup combinations placed the lowest importance on the part that the complexity of the *Navigation Pathway* in an airport plays in having an impact on their ability to find their way around an airport effectively. For the *Female, Personal, Inexperienced* group, this importance is placed on the level of *Human Factors*, whereas the *Male, Business, Experienced* group placed this importance on the effectiveness of the *Visual Elements of Communication*. Both subgroups had a very small spread of responses for *Environmental Factors*. Interestingly, the spread of responses for *Wayfinding* was quite different for both subgroups with the *Males, Business, Experienced* group having a wider spread in responses.

## 6. Conclusions

As discussed and shown in the previous sections, prior and posterior pooling have both advantages and disadvantages. Here, we summarise these, especially in the interest of those who should choose between them in practical situations.

PrLP is the fastest way to undertake the analysis of a BN because the expert opinions are pooled and placed into a single BN. Because of this, diagnostic and predictive reasoning, scenario testing, and what-if analysis are easier to undertake. Additionally, processes like sensitivity analyses are easy to perform, as there is only one BN to contend with and the popular BN software can do this easily. Disadvantages of this method are that the resulting BN does not follow a coherent probability model, in the sense that one can think of each expert’s assessment as his/her estimator of the probability based on his/her own model, whereas no such model is behind the estimator obtained when pooling the opinions. Practically, since the probabilities given by each expert are pooled within each entry in the CPT, these probabilities are not a reflection of the information that was originally obtained from the expert. As such, the conditional independence structure of the network is lost. This issue could be addressed to some extent by undertaking a validation process similar to that proposed by [37]. This approach is based on psychometric testing and includes nomological, face, content, concurrent, predictive, convergent and discriminant validity. A network model that passes all seven tests can be deemed to be empirically coherent.

Another disadvantage of the PrLP method is that information regarding the descriptive statistics for the responses provided, particularly if responses of subgroups are of interest, can not be easily obtained. Gaining information on the spread, distribution and mean of the responses from subgroups is difficult. PoLP, on the other hand, is able to provide this kind of information.

Posterior Linear Pooling can provide valuable information about the responses from experts. As has been shown, information regarding the mean, standard deviation, and the spread of subgroup responses to the nodes of interest can be obtained when using PoLP. This analysis can show behaviors from the subgroups that may not be as obvious if PrLP is being used. Additionally, this method ensures that the conditional independence structure of the BN is maintained since pooling occurs at the ‘end’ of the process. Of course, the main disadvantage to PoLP comes from having to construct a BN for each expert. Posterior Linear Pooling is more time consuming as it requires the handling of every BN in the problem, which in this case study was 99 BNs. If there is a large number of BNs involved, it will take more time to update the information in the network, undertake predictive and diagnostic reasoning, and investigate scenario and what-if analyses. A similar computational problem arises when the number of nodes in the BN increases. Notwithstanding this, the additional steps of the PoLP method and the associated computational burden may not be too onerous since polynomial inference algorithms only impose an additional O(n) factor in the computational complexity. Moreover, parallel programming with associated automatic updating can reduce the burden on the reasoning steps.

Regarding the application of these methods to the WBNM, both PrLP and PoLP found that *Gender* and *Travel Experience* have the greatest impact on *Human Factors* and *Wayfinding*. PoLP was also able to show that the combination of *Female*, *Personal*, *Inexperienced* travelers placed the greatest importance on *Human Factors*, whereas *Male*, *Business*, *Experienced* placed more importance on *Visual Elements of Communication*. *Inexperienced* travelers were also found to require higher probabilities of *Effective Communication* and *Good Environmental Factors* in order to easily find their way around the airport. This, of course, is not a surprising result. Of the six nodes of interest, the complexity of the *Navigation Pathway* was one that was not important to the subgroups investigated.

This investigation has some implications for airport operators, which back up the results found by Farr et al. [12]. That is, Human Factors have a large impact on effective wayfinding in airports, more so than the other nodes that were investigated in this paper. This means that the airport environment has to be designed to allow human factors to be ‘Good’. Additionally, the catering to the Inexperienced Traveler has to be a consideration. As shown, this subgroup is the one that has the largest difference in outcomes across all the nodes of interest that were investigated. Examples of how airports can cater to inexperienced travelers could include providing airport and travel process information online, and at the time of booking.

## Figures and Tables

**Table 1:** Advantages and disadvantages of using Prior Linear Pooling and Posterior Linear Pooling for combining the opinions of multiple experts.

Method	Advantages	Disadvantages
Prior Linear Pooling (PrLP)	· Smaller number of steps are required to obtain marginal probabilities of interest. · Having only one BN makes updating information easier and more timely. · Diagnostic, predictive, and intercausal reasoning are easier to undertake.	· Pooling, when used with BNs do not follow a coherent probability model [25]. · Since each of the probabilities given by the experts are pooled within each entry of the CPT, the resulting averages are not a reflection of what was originally given by the expert for that entry, and so the conditional independence structure is lost.
Posterior Linear Pooling (PoLP)	· The conditional independence structure of the BN is maintained.	· More steps are required in order to obtain the marginal probabilities of interest. · Updating information can be time consuming if there are a large number of experts, and hence BNs. · Diagnostic, predictive, and intercausal reasoning is also time consuming if there are a large number of experts. This is because each individual BN must be modified and then pooling once again done to obtain the marginal probabilities of interest.

**Table 2:** The six nodes of interest, their definitions and respective states, as found in Farr et al. [36].

Node	Description	States
Communication	The effectiveness of communication in the airport terminal	Effective, Ineffective
Environmental Factors	The level of the environmental factors such as terminal design and navigation pathway complexity that contribute to effective wayfinding in airport terminals	Good, Bad
Human Factors	The level of the human factors such as spatial anxiety and cognitive and spatial skills that contribute to effective wayfinding in airport terminals	Good, Bad
Navigation Pathway	The complexity of the navigation pathway that a passenger must traverse in order to reach a desired destination in the airport terminal	Simple, Complex
Visual Elements of Communication	The quality of the visual elements of communication in the airport terminal	Good, Bad
Wayfinding	The effectiveness of wayfinding in the airport terminal	Effective, Ineffective

**Table 3:** The marginal probabilities for the *Human Factors* and *Wayfinding* nodes, using Prior Linear Pooling, for the full network and the three subgroups Gender (Female, Male), Travel Purpose (Business, Personal), and Travel Experience (Experienced, Inexperienced). Since the nodes have binary states, the probability for one state per node is shown.

Group	Human Factors Good	Wayfinding Effective
All	0.8033	0.8057
Female	0.7790	0.7876
Male	0.8369	0.8305
Business	0.8033	0.8057
Personal	0.8033	0.8057
Experienced	0.8135	0.8132
Inexperienced	0.7458	0.7683

**Table 4:** The marginal probabilities for *Human Factors* and *Wayfinding* for the full BN, as well as for combinations of the subgroups *Gender* and *Travel Experience*. The absolute value of the differences between the full model and each subgroup is shown in the square brackets.

	Good Human Factors	Effective Wayfinding
All	0.8033	0.8057
Female, Experienced	0.7880 [0.0153]	0.7943 [0.0114]
Female, Inexperienced	0.7282 [0.0751]	0.7500 [0.0557]
Male, Experienced	0.8487 [0.0454]	0.8393 [0.0336]
Male, Inexperienced	0.7701 [0.0332]	0.7810 [0.0247]

**Table 5:** Marginal probabilities for diagnostic reasoning for the WBNM. Changes in the nodes of interest are shown when the *Wayfinding* node is set to be 100% effective. The absolute value of the differences between the full model and each subgroup is shown in the square brackets. The comparison shown is made between the full model with the *Wayfinding* node at 100% *Good*, and then each node chosen at 100% for each subsequent subgroup.

Group	Communication Effective	Environmental Factors Good	Human Factors Good	Navigation Pathway Simple	Visual Elements of Communication Good
Full network	0.8115	0.7697	0.8033	0.6893	0.7087
Female	0.8183 [0.0068]	0.7931 [0.0234]	0.9410 [0.1377]	0.6941 [0.0048]	0.7114 [0.0027]
Male	0.8239 [0.0012]	0.7901 [0.0020]	0.9587 [0.1554]	0.6935 [0.0042]	0.7137 [0.0050]
Business	0.8207 [0.0092]	0.7918 [0.0021]	0.9487 [0.1454]	0.6939 [0.0046]	0.7124 [0.0037]
Personal	0.8207 [0.0092]	0.7918 [0.0021]	0.9487 [0.1454]	0.6939 [0.0046]	0.7124 [0.0037]
Experienced	0.8201 [0.0086]	0.7947 [0.0022]	0.9518 [0.1485]	0.6937 [0.0044]	0.7121 [0.0034]
Inexperienced	0.8246 [0.0013]	0.7947 [0.0025]	0.9300 [0.1267]	0.6945 [0.0052]	0.7139 [0.0052]

**Table 6:** The marginal probabilities for the six nodes of interest, using Posterior Linear Pooling, for the full network and the three subgroups Gender (Female, Male), Travel Purpose (Business, Personal), and Travel Experience (Experienced, Inexperienced). The absolute value of the differences between the full model and each subgroup is shown in the square brackets. Since the nodes have binary states, the probabilities for one state per node is shown.

Group	Communication Effective	Environmental Factors Good	Human Factors Good	Navigation Pathway Simple	Visual Elements of Communication Good	Wayfinding Effective
All	0.7415	0.7672	0.7082	0.6509	0.8188	0.7517
Female	0.7430 [0.0014]	0.7680 [0.0007]	0.7400 [0.0318]	0.6524 [0.0015]	0.8194 [0.0006]	0.7546 [0.0029]
Male	0.7403 [0.0012]	0.7666 [0.0006]	0.6811 [0.0270]	0.6495 [0.0013]	0.8182 [0.0005]	0.7492 [0.0024]
Business	0.7413 [0.0002]	0.7674 [0.0001]	0.6698 [0.0383]	0.6517 [0.0008]	0.8206 [0.0018]	0.7585 [0.0067]
Personal	0.7416 [0.0001]	0.7672 [0.00006]	0.7247 [0.0164]	0.6505 [0.0003]	0.8180 [0.0007]	0.7488 [0.0029]
Experienced	0.7363 [0.0052]	0.7666 [0.0006]	0.7006 [0.0076]	0.6503 [0.0005]	0.8180 [0.0007]	0.7485 [0.0031]
Inexperienced	0.7690 [0.0274]	0.7705 [0.0032]	0.7482 [0.0399]	0.6537 [0.0028]	0.8230 [0.0041]	0.7683 [0.0165]
